# Extending the CARE Principles from tribal research policies to benefit sharing in genomic research

**DOI:** 10.3389/fgene.2022.1052620

**Published:** 2022-11-11

**Authors:** Stephanie Russo Carroll, Rebecca Plevel, Lydia L. Jennings, Ibrahim Garba, Rogena Sterling, Felina M. Cordova-Marks, Vanessa Hiratsuka, Maui Hudson, Nanibaa’ A. Garrison

**Affiliations:** ^1^ Mel and Enid Zuckerman College of Public Health, University of Arizona, Tucson, AZ, United States; ^2^ Native Nations Institute, Udall Center for Studies in Public Policy, University of Arizona, Tucson, AZ, United States; ^3^ Law Library, School of Law, University of South Carolina, Columbia, SC, United States; ^4^ Te Kotahi Research Institute, University of Waikato, Hamilton, New Zealand; ^5^ Center for Human Development, College of Health, University of Alaska Anchorage, Anchorage, AK, United States; ^6^ Institute for Society and Genetics, College of Letters and Sciences, University of California, Los Angeles, Los Angeles, CA, United States; ^7^ Institute for Precision Health, David Geffen School of Medicine, University of California, Los Angeles, Los Angeles, CA, United States; ^8^ Division of General Internal Medicine & Health Services Research, David Geffen School of Medicine, University of California, Los Angeles, Los Angeles, CA, United States

**Keywords:** research policy, indigenous, data sovereignty, data governance, care principles

## Abstract

Indigenous Peoples have historically been targets of extractive research that has led to little to no benefit. In genomics, such research not only exposes communities to harms and risks of misuse, but also deprives such communities of potential benefits. Tribes in the US have been exercising their sovereignty to limit this extractive practice by adopting laws and policies to govern research on their territories and with their citizens. Federally and state recognized tribes are in the strongest position to assert research oversight. Other tribes lack the same authority, given that federal and state governments do not recognize their rights to regulate research, resulting in varying levels of oversight by tribes. These governance measures establish collective protections absent from the US federal government’s research oversight infrastructure, while setting expectations regarding benefits to tribes as political collectives. Using a legal epidemiology approach, the paper discusses findings from a review of Tribal research legislation, policy, and administrative materials from 26 tribes in the US. The discussion specifies issues viewed by tribes as facilitators and barriers to securing benefits from research for their nations and members/citizens, and describes preemptive and mitigating strategies pursued by tribes in response. These strategies are set within the framing of the CARE Principles for Indigenous Data Governance (Collective Benefit, Authority to Control, Responsibility, Ethics), a set of standards developed to ensure that decisions made about data pertaining to Indigenous communities at the individual and tribal levels are responsive to their values and collective interests. Our findings illustrate gaps to address for benefit sharing and a need to strengthen Responsibility and Ethics in tribal research governance.

## 1 Introduction

Indigenous Peoples have historically been targets of extractive research, approaches that not only expose communities to the risks of misuse (e.g., stigmatizing findings, stereotyping), but also deprive them of potential benefits (e.g., information on disease risk factors, preventative care, therapies, safety). Tribes in the US have been exercising their sovereignty to limit extractive research practices by adopting laws, policies, and processes governing research on their territories and citizens, including setting expectations regarding benefits to tribes as political collectives.

This paper extends the authors’ work on Indigenous Peoples’ expectations regarding genomic research (Garrison et al., 2019; Hudson et al., 2020) and how tribes set research and data expectations through codes and policies (Hiraldo et al., 2021; Carroll et al., 2022). Using legal epidemiology, the study and deployment of law as a factor in the cause, distribution, and prevention of harm and injury in a population (Burris et al., 2016), we describe how research legislation, policy, and processes from 26 tribes in the US shape benefit sharing expectations in research. The discussion identifies policy gaps for securing benefits from research and describes preemptive and mitigating strategies pursued by tribes. These strategies align with the CARE Principles for Indigenous Data Governance (Collective Benefit, Authority to Control, Responsibility, Ethics), standards developed to ensure that decisions about Indigenous data are responsive to tribal values and interests (Carroll et al., 2020). We close with proposed measures tribal rights holders can enact and strategies others (e.g., sponsors/funders, research entities, scientists) can use to advance ethical benefit sharing with Indigenous communities.

## 2 Benefit sharing in indigenous research

The concept of benefit sharing emerged from the Nagoya Protocol on Access to Genetic Resources and the Fair and Equitable Sharing of Benefits Arising from their Utilization to the Convention on Biological Diversity (United Nations, 2010) to describe the need to share benefits arising from extraction of natural resources with local communities (Petrov and Tysiachniouk, 2019). The discourse around benefit sharing combines elements of international law, research ethics, and political philosophy, and its primary application was to genetic resources, mining, and international clinical research, although it is used in much wider contexts now (Dauda and Dierickx, 2013). Benefit sharing is mostly ignored in research despite being regarded as an ethically sound concept (Lairumbi et al., 2012). Historically, Indigenous communities have been subjected to extensive research with little input regarding the research process, study results, or decision-making about how those data are disseminated in both academic and community settings (Smith, 2012). Tribal sovereignty, research oversight, and international Indigenous-led standards address past challenges and expand considerations of how research benefits Indigenous nations.

### 2.1 Harms of extractive research

To understand the range of extractive research practices, we briefly describe examples from genomics and environmental sciences which inform Indigenous Peoples’ interest and reluctance to participate in research. It is important to understand these perspectives to advance Indigenous-led and Indigenous-supported research that utilizes Indigenous research and data governance. First, harms from Indigenous participation in research have far outweighed benefits, as exemplified in the cases of genetic research by Arizona State University with the Havasupai Tribe and the University of British Columbia with the Nuu-chah-nulth First Nation (Wiwchar, 2004; Drabiak-Syed, 2010)*.* Both tribes experienced a multitude of harms, with one case resulting in a lawsuit, and raised concerns about exploitation, lack of engagement, and group harms in genetic research (Garrison et al., 2019).

Second, research involving Indigenous Knowledges in environmental sciences has grown over 200% since the 1990s (Carter et al., 2020; Jessen et al., 2022). Non-Indigenous scientists have often conducted research on Indigenous lands without community involvement or expertise (Minasny et al., 2020; Native Nations Institute, 2022). Researchers benefit from local infrastructure and knowledge but do not value local scientists or knowledge holders as equal partners, sharing minimal benefit with communities hosting the research (Adame, 2021; Handsley-Davis et al., 2021; Nature, 2022).

### 2.2 Emergence of Indigenous oversight

#### 2.2.1 Tribal research regulation

Over the past 50 years, tribes in the US have developed and implemented policies and procedures for research oversight within their nations’ territories and beyond. Some tribes rely on tribal colleges, tribally-based organizations, or the Indian Health Service to provide research oversight on their behalf (Around Him et al., 2019). While federally and state recognized tribes are in the strongest legal position to assert authority over their data (Tsosie, 2019), non-recognized tribes and other Indigenous Peoples also expect the recognition of their rights in data to translate into opportunities for benefit. We posit that tribal sovereignty, research oversight, and Indigenous-led research can address known challenges and expand consideration of how research can benefit these nations.

US research is governed according to the Federal Policy for the Protection of Human Subjects that was influenced by the 1979 Belmont Report written by the National Commission for the Protection of Human Subjects of Biomedical and Behavioral Research. The Belmont Report hinges on the principles of respect for persons, beneficence, and justice. Saunkeah et al. (2021) describe the Belmont Report’s shortcomings around reciprocity and lack of community focus. Garrison et al. (2019) compared research policies for US, Canada, New Zealand, and Australia to the Indigenous Research Protection Act, a document for tribal leaders and attorneys to consult for strengthening research governance within tribal nations. Shortfalls of US policy present challenges for Indigenous Peoples as well as opportunities for innovation to support equitable and beneficial research.

The call for community-driven and engaged research and data practices began over 20 years ago (Mauro and Hardison, 2000; Arbour and Cook, 2006; Minkler and Wallerstein, 2008; Wallerstein et al., 2018). More recently, Indigenous researchers have developed frameworks, drawing on Indigenous values and customs, to ethically engage communities in research (Claw et al., 2018; David-Chavez and Gavin, 2018; Ward et al., 2020). The National Institutes of Health (NIH) finalized a data management and sharing policy in 2020 based on themes that emerged from tribal consultation processes. The policy clarifies agency respect for tribal sovereignty in the absence of written tribal laws or policies while also recognizing that tribal nations may wish to manage, preserve, and share their own data. The NIH supports efforts that enable Native communities to prioritize research opportunities and to ensure sufficient protections of scientific data generated.

#### 2.2.2 International standards for indigenous research

The United Nations Declaration on the Rights of Indigenous Peoples (UNDRIP) affirms various rights to be acknowledged and pursued in a spirit of partnership and mutual respect (United Nations, 2007). The Nagoya Protocol is an international agreement supporting equitable sharing of benefits from the utilization of genetic resources. It came into effect in 2014, building on the requirement for mutually agreed terms as well as access and benefit sharing established through the Convention on Biological Diversity (United Nations, 2011). While International standards were being negotiated, Indigenous collectives also developed frameworks to assert their rights e.g., the Mataatua Declaration on Cultural and Intellectual Property Rights of Indigenous Peoples and the First Nations Principle of OCAP^®^ (a registered trademark of the First Nations Information Governance Centre) that details Ownership, Control, Access, and Possession (FNIGC, 2018).

Since 2015, Indigenous Data Sovereignty (IDSov) has emerged in scholarship and practice worldwide (Kukutai and Taylor, 2016a; Rodriguez-Lonebear, 2016; Rainie et al., 2017; Chung and Chung, 2019; Rainie et al., 2019; Walter et al., 2021). Indigenous data, whether born digital or not, include information and knowledge (also specimens and material belongings) about Indigenous Peoples at the individual or collective levels (Lovett et al., 2019; Rainie et al., 2019). IDSov maintains that Indigenous Peoples hold authority over data about their nations, citizens, communities, and non-human relations, regardless of the location of those data (Kukutai and Taylor, 2016b; Carroll et al., 2022).

The CARE Principles were developed to complement the FAIR principles for scientific data management (Findable, Accessible, Interoperable, Reusable) (Wilkinson et al., 2016; Carroll et al., 2019; Carroll et al., 2020). Operationalizing FAIR with CARE guides the collection, use, and sharing of Indigenous data to align with Indigenous rights and interests in the age of big data and open science (Carroll et al., 2021). The CARE Principles are being implemented by nation-states and major institutions (e.g., UNESCO Recommendation on Open Science, the AIATSIS Code of Ethics for Aboriginal and Torres Strait Islander Research, the Aotearoa New Zealand Antarctic and Southern Ocean Research Directions and Priorities). Together, these frameworks anticipate benefit sharing with Indigenous Peoples in the use of derived genetic resources and traditional knowledge. Here, we use the CARE Principles to illustrate tribal expectations for benefit sharing.

## 3 Tribal expectations for research benefits

Using legal epidemiology (also known as “policy surveillance”) and grounded theory to analyze the content of tribal research governance documents, we conducted an inductive analysis of tribal legislation and policy (Burris et al., 2016). Presenting a set of rights and expectations, these governance documents help ensure that researchers and institutions propose and conduct genomic and other research that do not denigrate or harm Indigenous rights, knowledges, or health.

We reviewed documents from 26 US tribal communities, including: 1) legislation (e.g., acts, codes, ordinances, regulations); 2) policies, procedures, protocols; 3) application overview/background documents; 4) application guidance; and 5) application materials (e.g., forms, templates) (Table 1). These materials are living documents, and legal epidemiology requires an end date for collection, which, in this case, is June 2021. Some tribes have since updated their materials. We masked tribe names to maintain anonymity in the resulting tables and discussion. One tribe’s document(s) had no relevant content for analysis. Table 2 distills the provisions discussing community and individual benefits. We draw on regulations from federally and state recognized tribes to illustrate how these official documents address the benefits (and risks) of research (Supplementary Table S1) and set tribal expectations for commercialization, publication, collaboration, and shared authorship (Supplementary Table S2). We present these tribal expectations using the CARE Principles to inform tribes as they design and revise governance mechanisms, and as guidance for institutions as they implement laws and policies to support Indigenous Peoples’ data and research rights and interests (Table 2).

**TABLE 1:** Tribal research governance documents reviewed.

Tribe	Type of documents reviewed				
	Legislation (act, code, ordinance, regulation, resolution)	Policy/procedure/protocol	Application overview/background	Application guidance	Application materials
Aleut Community of St. Paul Island	1				
Cherokee Nation			3	5	2
Eastern Band of Cherokee Indians			1	3	2
Chickasaw Nation		6			5
Colorado River Indian Tribes	1				3
Confederated Tribes of the Colville Reservation	1		1		1
Confederated Tribes of Coos, Lower Umpqua and Siuslaw Indians	1				
Confederated Tribes of Siletz Indians	1				
Gila River Indian Community	1	2		4	1
Ho-Chunk Nation	2				
Hopi Tribe		1	1		
Karuk Tribe		2			
Mandan, Hidatsa, and Arikara Nation (Three Affiliated Tribes)	1				
Muscogee (Creek) Nation	1				
Navajo Nation	1	1	1	3	3
Nez Perce Tribe	1				1
Oglala Sioux Tribe			3		
Pascua Yaqui Tribe	1				
San Carlos Apache Tribe		1			
Seneca Nation of Indians		1			
Sisseton Wahpeton Oyate of the Lake Traverse Reservation	1			1	
Saint Regis Mohawk Tribe		1			
Tohono O’odham Nation	1				
Turtle Mountain Band of Chippewa Indians	1			2	1
United Houma Nation	1				
White Earth Band of the Minnesota Chippewa Tribe	1	1			

**TABLE 2:** Operationalizing the CARE principles: Tribal expectations for benefit sharing.

CARE principle	Issues raised by communities	Actions for institutions and researchers
Collective benefit		
	Compensation	Compensation should not be viewed merely as monetary payment for participation in studies, but should be understood to include access to research findings; acknowledgment as author, co-author, or contributor; intellectual property rights; employment and training; and technical assistance.
	Royalties	Tribal entities must be consulted, and contracted with, for the provision of and/or the division of royalties from any products of research.
	Benefits of research generally	The relevance and value of research results to the interests of the Tribe and individual members must be explained and demonstrated.
	Collective benefit	The intent of the research project as well as the benefit(s) that the project, research, or activity will bring to the Tribal community (in addition to researchers and funders) should be clearly explained.
	Lack of benefit	Prior to obtaining permission to conduct studies with Tribes and their members, it should be demonstrated that the project will not be purely extractive, but will be collaborative and inclusive.
	Health	Health-related research should directly benefit the Tribal collective and individual members, and explanations should be given for how study results will be used to improve the health status of the Tribe and its members.
Authority to control		
	Commercialization	A separate agreement will be made if research outcomes are to be commercialized, as agreed to by the Tribal entity. Restrictions on commercialization or profit can be made.
	Intellectual property	Tools for transparency, integrity, and provenance (e.g., legal agreements, permits, licensing, authorship) should be used.
	Return of/access to findings	Results and findings from research with Tribal entities and communities must first be provided to those nations, communities, and individuals, and the appropriate Tribal entities must be provided with all final reports and data.
	Authorship and acknowledgement	Tribal contributions to the study/project must be acknowledged and properly credited by recognition of individuals and collectives involved in the development, implementation, and return of findings, at the discretion of the Tribe, group, and individuals.
Responsibility		
	Empowerment	Tribal ideas, values, and voices must be included in the research process, including language and lived experiences.
	Employment/training	Beyond participation in the study, Tribal members should be employed by the project, trained to conduct such research, and be involved in the dissemination of findings.
	Technical assistance	Assistance should be available to the Tribe for writing grants, conducting in-service trainings, developing educational materials, planning Tribal research conferences, and obtaining equipment.
Ethics		
	Benefit-risk balance	Studies and research projects must present only reasonable risks in relation to anticipated benefits to the Tribe and its members, as determined by the community.

Tribal expectations within these documents coalesce around five themes: 1) Benefits of research (generally); 2) Collective (i.e., tribal, community) benefit/value/relevance/interest; 3) Historical and continuing lack of benefit; 4) Balancing benefits and risks/harms; and 5) Restrictions on/prohibitions of commercialization/profit (Supplementary Table S1). Many of the codes and policy documents delineate an equitable distribution between the Tribe and the researcher, both in treatment of participants and sharing of credit and research results.

Benefits described within tribal research governance documents fall into two categories: 1) Economic and 2) Self-determination, capacity sharing, and community building (Supplementary Table S2). Economic benefits comprise 1) Compensation, 2) Value from commercialization/profit, 3) Royalties, and 4) Intellectual property. Benefits around Self-determination, capacity sharing, and community building include 1) Return of/access to findings, 2) Authorship and acknowledgement/credit in publications, 3) Empowerment, 4) Employment/training, 5) Technical assistance, and 6) Health.

Economic benefits are an avenue for meaningful benefits to be shared with the community. Tribal codes describe compensation, value from commercialization, royalties, and intellectual property (IP) as key areas for discussion with researchers. While there is not always a clear distinction between categories, as some tribes refer to royalties and IP in the context of compensation, economic benefits are important sites of negotiation. However, given the variety in types of research, economic benefits are one of the most challenging to generate tangible results consistently.

## 4 Issues and actions for implementing CARE

Using the CARE Principles, Table 2 details the thematic areas in which tribes address benefit sharing within their tribal research codes, policies, and processes.

### 4.1 Collective benefit

Collective benefit is core to IDSov and reinforces rights to engage in decision-making according to Indigenous values and collective interests. It focuses on inclusive development and innovation, improved governance and citizen engagement, and equitable research outcomes. Value generation is an important element in enabling collective benefit with expectations for sharing with the community; examples can be economic (such as compensation and royalties) or focused on health. As moral and legal responsibility to support benefit sharing has evolved, so has the differentiation between monetary and non-monetary benefits. Research that aims to offer benefits often focus on hiring or authorship practices and less on the sharing of IP, derivative products, or supporting tribal sovereignty and Indigenous-led research.

Tribes have a right to expect appropriate *compensation* for participation in research activities, including being paid as research participants, informants, and/or consultants, as well as other benefits such as co-authorship, sharing of copyright, and training or education expenses (Tribes 3, 5, 10, 18, 24). Some tribes charge fees for research permits or licenses (Tribes 1, 2, 4, 8, 12, 14, 18, 20, 21, 23, 24, 25). Tribe 3 requires compensation “for research-related inconveniences,” while others ask for “just compensation or fair return” (Tribes 18, 25). Depending on the project, compensation is often the only pathway for economic benefits to tribal communities. However, some tribal codes explicitly request royalties from products derived from Indigenous peoples, culture, lands, and biological resources (Tribes 6, 12, 13, 18, 20, 24, 25). The tribal codes do not specify the nature of the royalty but highlight the need to plan for potential royalties and write royalty clauses into agreements (Tribes 12, 24).

The documents distinguish how benefits need to be assessed or measured at the collective (i.e., tribal and community) level, in contrast to the Belmont Report’s focus on the individual (Tribes 1, 3, 5, 6, 7, 9, 10, 11, 13, 15, 16, 18, 22, 25, 24). Collective benefits have value, relevance, and are of interest to the tribe and tribal community. The documents also include research benefits to address past and continuing experiences for projects that were not designed to provide any benefits (Tribes 2, 3, 4, 11, 15). A few also note ways that tribes are acknowledging and addressing this lack of benefit in research, ranging from denial of research proposals (Tribe 11) to approval of projects accompanied by a statement that no benefits are expected (Tribe 15). Rather than vaguely describing research benefits for the “public good” or “public interest,” some documents ask researchers to identify potential beneficiaries who are receiving benefits, or absorbing the costs, of research. Examples of beneficiaries are research participants, tribal members, families, tribes, Indigenous Peoples, society, researcher/investigator, project personnel, environment, science, and human knowledge (Tribes 1, 3, 4, 6, 7, 8, 9, 11, 13, 15, 16, 18, 19, 22, 23, 24, 25).

Finally, tribal documents highlight the importance of addressing health and well-being in research, prompting some tribes to require activities be respectful while promoting health in ways that are aligned with healthy lifestyles for all generations (Tribes 1, 11, 15).

### 4.2 Authority to control

Conducting research with tribal communities involves supporting capacity sharing, community partnership building, self-determination, and tribes’ rights to govern research on their lands, with their peoples, and their non-human relatives. Authority to control appears in the contexts of commercialization (including restrictions), IP, return of and access to findings, and authorship and acknowledgment.

Commercialization involves turning research into viable profit-earning products. Some codes restrict, or require specific applications and permissions for, commercialization (Tribes 4, 18, 24). Some require separate commercial agreements (Tribe 24), while others establish an obligation to share financial benefits from commercialization (Tribe 12). In contrast, some research agreements include a statement of noncommercial use of research products (Tribe 4).

IP such as patents, copyright, and trademarks are valuable assets. In past extractive research with Indigenous Peoples, researchers have not recognized tribal rights to data. Tribes are now recognizing this lack of input into the research process (Smith, 2012) and are adopting policies and laws to protect their IP, assert their Cultural Intellectual Property Rights, and ensure their cultural authority is recognised within records (Golan et al., 2022). These measures set expectations that tribal control of knowledge will be maintained by exclusive assignment or negotiation of applicable IP rights (Tribes 6, 10, 12, 13, 14, 18, 24, 25). For example, some codes affirm IP claims to “cultural, linguistic, and historic information” not otherwise belonging to the researcher (Tribe 14), and others establish a process for negotiating “ownership of copyrights” (Tribe 13).

Return of, or creating access to, research findings recognizes that tribes have the right to access their data and the authority to govern future use. Tribal research documents also define return of, and access to, research findings as creating opportunities for co-authorship or receiving acknowledgement and proper credit for their contributions (Tribes 5, 6, 10, 12, 15, 24, 25).

Tribes have increasingly asked to be credited for their contributions in publications as co-authors or to have the tribe/community members acknowledged. Several research codes have clarified this by describing co-authorship as reciprocity (Tribe 10) or by requiring that the research applicant spell out “how the Tribal community” will “share in the authorship” of the research (Tribe 24). Several journals (*Rural and Remote Health*, the *Australian Journal of Rural Health*, and the *Canadian Journal of Rural Medicine, Data Science Journal*) have now made Indigenous co-authorship a requirement for publication of Indigenous related content (Lock et al., 2022).

### 4.3 Responsibility

Responsibility centers on forming positive relationships between researchers or data stewards and Indigenous Peoples by enhancing capacity and capability and by prioritizing Indigenous languages and world views. Tribal codes address this responsibility through requirements for empowerment, employment and training, and technical assistance.

Empowerment refers to equitable sharing of decision-making power so that tribes can influence how research is conducted, what knowledge is shared, and who is involved; it also includes supporting opportunities for partnerships, employment, and training (Tribes 6, 10, 11, 24). Tribal documents have cited the importance of “shared power, shared resources and mutual understanding” (Tribe 25) arising from “a good research agreement” that supports respect and equity (Tribe 6).

Conducting research in tribal communities requires a team to support all aspects of data collection, analysis, and dissemination. Opportunities are ripe for offering training to community members (e.g., students, aspiring researchers) to strengthen their research skills and for budgeting to hire qualified community members to assist on projects (Tribes 1, 6, 7, 10, 18, 20, 25). Some tribes ask researchers to offer first preference of employment to qualified tribal members (Tribes 18, 20). Sometimes, supporting tribal capacity and infrastructure means offering technical assistance for grant writing, training, developing educational materials, or supporting local libraries, archives and museums (Tribes 1, 10, 25).

### 4.4 Ethics

Ethics grounded in Indigenous values drive the just determination of risks and benefits, and future uses of data. In addition to discussing the benefits of research to various constituents (individuals, the tribe, broader communities) and undertakings (tribal sovereignty, language, culture, and science) (Tribes 4, 6, 7, 8, 10, 12, 14, 15 16, 24, 22), some documents incorporate the balancing of benefits against risks or harms (Tribes 6, 14).

## 5 Conclusion

Notably missing from the documents examined were elements of Responsibility and Ethics. Responsibility in CARE relates to the institutional duty to use data to support Indigenous worldviews, which would operationalize Indigenous guidelines, standards and protocols within institutional policies and practice. Missing from tribal research governance documents are explicit requirements for benefit sharing that align with Indigenous world views. Likewise, ethical future use of data requires guidance on appropriate actions for researchers and institutions to ensure benefits accrued are shared equitably with Indigenous communities. Practices that support acknowledgment, attribution, and authorship enable pathways for appropriate access and authority to be recognized. It is important to include Indigenous metadata and perpetuate provenance, protocols, and permissions throughout the data lifecycle to ensure generation of benefits (Golan et al., 2022).

As outlined here, the CARE principles provide a useful framework for addressing both the shared aspirations of many communities, as well as reiterating the value and importance of research oversight for tribal nations. Operationalizing the CARE Principles will highlight areas to strengthen tribal research governance and make institutional policies and practices more responsive. Even for tribes lacking federal or state recognition, the CARE Principles set expectations for developing and sustaining beneficial research partnerships. The challenge is to translate expectations from tribal research governance into data infrastructures and across research ecosystems, a process that will enhance transparency and accountability, while creating greater opportunities for benefit sharing.

## Data Availability

The original contributions presented in the study are included in the article/Supplementary Material, further inquiries can be directed to the corresponding author.
